# Acquired Epidermodysplasia Verruciformis in Patients with Iatrogenic Immunosuppression

**DOI:** 10.3390/jcm15052049

**Published:** 2026-03-07

**Authors:** Neha S. Momin, Peter L. Rady, Stephen K. Tyring

**Affiliations:** 1 Tilman J. Fertitta Family College of Medicine, University of Houston, Houston, TX 77021, USA; 2 Department of Dermatology, The University of Texas McGovern Medical School, Houston, TX 77030, USA

**Keywords:** epidermodysplasia verruciformis, immunosuppression, solid organ transplant, iatrogenic immunosuppression, beta human papillomavirus, squamous cell carcinoma

## Abstract

**Background:** Acquired epidermodysplasia verruciformis (AEV) is a rare cutaneous disorder arising in immunocompromised individuals. AEV is characterized by flat-topped, wart-like, or hypopigmented lesions predominantly on sun-exposed areas. Unlike classic genetic EV, AEV develops in the absence of germline mutations or family history. AEV most commonly arises in patients receiving iatrogenic immunosuppressive therapy for organ transplantation, autoimmune disease, or hematologic disorders. **Methods:** A comprehensive literature review was conducted via the PubMed database. Case reports and case series studies describing AEV in transplant and non-transplant iatrogenic immunosuppression were identified through a literature search. There were no restrictions on language or publication year. The last search was conducted in July 2025. Reports were analyzed for patient demographics, immunosuppressive agents, HPV subtypes, clinical and histopathologic features, and treatment outcomes. **Results:** AEV occurs across a broad spectrum of immunosuppressive therapies, including calcineurin inhibitors, antimetabolites, biologics, tyrosine kinase inhibitors, and cytotoxic chemotherapy. β-HPV subtypes, most commonly HPV 5 and 8, drive lesion formation in the context of impaired cell-mediated immunity. Histopathology demonstrates keratinocyte vacuolization, acanthosis, and perinuclear halos. Lesions may persist despite immunosuppressive adjustment, due to viral latency and incomplete immune reconstitution. Treatment strategies are varied and include topical retinoids, immune response modifiers, systemic retinoids, and HPV vaccination, and have variable efficacy. AEV carries an elevated risk of cutaneous squamous cell carcinoma, particularly in transplant recipients, and highlights the need for proactive dermatologic management. **Conclusions:** AEV represents a clinically significant consequence of immunosuppression mediated by β-HPV. Early recognition, monitoring for malignant transformation, and individualized multimodal therapy are critical. Future studies should evaluate targeted interventions to enhance antiviral immunity and establish standardized treatment guidelines.

## 1. Introduction

Epidermodysplasia verruciformis (EV) is a rare skin condition characterized by multiple flat, wart-like or tinea versicolor-like lesions distributed along sun-exposed cutaneous surfaces, such as the trunk, extremities, and genitals [1,2]. HPV plays a central role in the pathophysiology of EV. The virus infects epithelial cells and induces hyperproliferative skin lesions which may be benign or possess malignant potential. There are EV-associated HPV subtypes (HPV 3, 5, 8, 9, 10, 12, 14, 15, 17, 19–25, 28, 29, 36, 46, 47, 49, and 50), which can be found within keratinocytes and are considered diagnostic markers [3,4].

EV is classified through whether it is present due to genetic predisposition or through an acquired form. Genetic EV can be broadly divided into classic forms driven by EVER1/EVER2/CIB1 mutations and non-classic forms associated with broader immunodeficiency syndromes. Classic genetic EV is inherited through an autosomal recessive pattern and is associated with mutations in the EVER1/TMC6, EVER2/TMC8, or CIB1 genes, which play an essential role in cell-mediated immunity against human papillomavirus (HPV) [5,6]. The EVER genes are transcribed in the CD4+ and CD8+ T-lymphocytes, B-lymphocytes, and natural killer cells at elevated levels, which suggests they play a role in immune response against HPV-related EV. These mutations impair the host’s ability to clear specific cutaneous HPV subtypes, allowing for persistent viral infection and lesion development. EVER genes also form a complex with zinc transporters (Zn-T1). The complex provides resistance against zinc toxicity and allows for zinc effusion, which acts as a form of anti-microbial resistance against HPV in unaffected individuals.

Non-classic genetic EV mutations are associated with the RHOH, MST-1, CORO1A, ARTEMIS, DOCK8, RASGPR1, LCK, and TPP2 genes [7,8]. Individuals with these mutations share similar features with classic genetic EV, such as having impaired immunity to HPV, predisposing them to EV, but also often present with additional diseases depending on the specific mutation type. Onset of both classic and non-classic genetic EV subtypes typically occurs in childhood, and patients remain chronically susceptible to HPV-related skin disease, including non-melanoma skin cancer.

More recently, reports of acquired EV (AEV) have been described in immunocompromised individuals without a family history of EV or genetic mutations [9]. In these patients, EV-like lesions appear later in life and arise secondary to impaired cell-mediated immunity, which is most commonly due to human immunodeficiency virus (HIV) infection or iatrogenic immunosuppression, including medications used in solid organ transplantation, biologic therapy, antimetabolites, tyrosine kinase inhibitors, or cytotoxic chemotherapy [7]. Histologically and clinically, AEV mirrors the classic form, but the pathogenesis is distinct and directly tied to immune dysfunction. Reports of patients receiving immunosuppressive therapy for non-transplant indications demonstrate that AEV is not exclusive to transplant recipients but reflects a broader consequence of sustained immune impairment.

The emergence of EV as a result of iatrogenic immunosuppression demonstrates a need to understand the pathogenesis of immunosuppressed individuals, how they acquire this condition, and how EV can be effectively managed. These patients not only develop characteristic EV lesions but also face an increased risk of HPV-related cutaneous malignancies. The need to identify strategies for early detection, prevention, and treatment is critical. This review aims to synthesize the current literature on the pathogenesis, clinical features, and treatment strategies of AEV in patients receiving iatrogenic immunosuppressive therapy. By better understanding the relationship between immunosuppression, HPV susceptibility, and EV development, effective preventative protocols and targeted strategies may be established across both transplant and non-transplant patient populations.

## 2. Materials and Methods

A comprehensive literature search was conducted to synthesize the existing literature on AEV occurring in the setting of iatrogenic immunosuppression, focusing on organ transplant recipients and non-transplant patients receiving immunosuppressive therapy. The search was conducted using the PubMed databases. The search included articles published through July 2025 and did not contain any date restrictions. Keywords used in the search included “epidermodysplasia verruciformis in transplant patients”, “epidermodysplasia verruciformis immunosuppression”, “acquired epidermodysplasia verruciformis”, and “acquired epidermodysplasia verruciformis human papillomavirus.” No language restrictions were applied, and translated versions of non-English publications were included when available. Reference lists of relevant publications were reviewed to identify additional eligible studies.

The initial search yielded 52 articles. Titles and abstracts were screened for relevance followed by full text review of potential studies. Inclusion criteria consisted of original research articles, case reports, case series, and review articles describing AEV in iatrogenic immunosuppressed patients or providing clinical, virologic, or therapeutic data. Articles focusing on inherited EV or cases of AEV occurring in the context of HIV-associated immunosuppression were excluded. HIV-related AEV was excluded, as HIV-related immunodeficiency involves distinct immunologic mechanisms compared to medication-induced immunosuppression, and the inclusion of these cases could confound interpretation of risk factors and treatment responses specific to iatrogenically immunosuppressed populations.

Included studies were organized using Zotero reference software (version 7.0.32) for citation management. Relevant data was extracted from included studies, including patient demographics, organ transplant type, immunosuppressive regimen and duration, indication for immunosuppression in non-transplant cases, associated HPV subtypes, treatment modality, and reported outcomes. Variables not reported in the original publications were recorded as “not reported” and were not imputed. Studies were then categorized into two groups: AEV following organ transplantation and AEV associated with non-transplant-related iatrogenic immunosuppression. Since most available studies were case reports or cases series, a formal risk of bias tool was not applied. Instead, qualitative descriptive synthesis was performed, and data was organized into tables to assess clinical, virologic, histopathologic, and treatment outcome reporting.

## 3. Human Papillomavirus Microbiology

**Figure 1:**
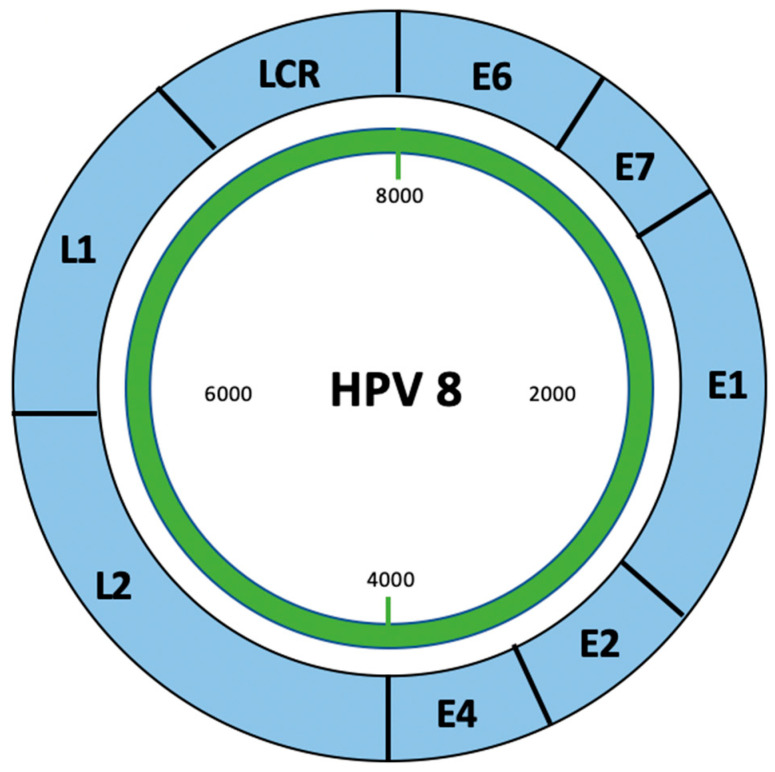
Genomic organization of β-HPV. Schematic representation of the β-HPV genome exemplified by HPV8. The genome consists of early (E), late (L), a non-coding region (NCR), and a long coding region (LCR). Early proteins regulate replication (E1, E2), viral release (E4), and oncogenesis (E6, E7), while late proteins (L1, L2) form the viral capsid. The LCR contains transcriptional elements essential for replication and tissue specificity.

The development of cutaneous warts in EV is associated with HPV, which is a small, non-enveloped, double-stranded DNA virus that belongs to the papillomaviridae family [10]. HPV has an icosahedral capsid composed of 72 capsomeres and circular DNA made up of 8000 base pairs. HPV is subdivided into ɑ and β genera. The β genus is associated with skin infections in immunocompromised patients and is typically seen in patients with EV. β-HPV lacks the E5 protein, which is present in α-HPVs [10,11,12,13]. E5 is known to enhance immune evasion and promote cell proliferation. The absence of the E5 protein in β-HPV suggests that these viruses rely more heavily on host immunosuppression rather than intrinsic viral mechanisms, which may explain why β-HPVs typically do not cause clinical disease in immunocompetent individuals [10]. β-HPVs have a less identifiable binding site for the E1 replication protein and contain shorter LCR, which suggests β-HPVs have different regulatory mechanisms compared to other HPV types (Figure 1) [10].

The life cycle of β-HPV starts when stratified epithelial cells become exposed to the virions through trauma. In order for β-HPV to enter the cell, there needs to be a heparan sulfate binding site on the host cell surface. Epithelial cells serve as ideal targets for β-HPV infection due to their ability to renew themselves, which allows the virus to survive for longer periods inside the host. The virus infects at the basal layer with E1 and E2 transcription and becomes independent from the cell cycle. The viral load is very low at the beginning, so the immune system is unable to detect the HPV infection [14]. E2 expression is amplified towards the upper layers with the progression of epithelial differentiation of the suprabasal and granular layers and is indirectly linked to the proliferation of keratinocytes. Unlike the high-risk α-HPV, β-HPV does not integrate into the host genome and instead is maintained as a viral episome [10]. Afterwards, the L1 and L2 proteins are expressed and participate in the assembly of virions, and E4 allows the β-HPV to be released for infection [14].

The β-HPV subtypes classically associated with EV patients are HPV 5 and 8. These subtypes have been frequently detected in cutaneous squamous cell carcinomas (SCCs) arising in transplant-associated EV patients. This finding suggests a higher oncogenic risk in the transplant population compared to immunocompetent individuals. Long-term immunosuppressive therapy compromises T-cell immunity, and antigen presentation allows viral life cycles of β-HPV types. β-HPVs are typically nonpathogenic in immunocompetent individuals but can persist and drive lesion formation [15]. The activity of the β-HPV LCR is regulated by E2 and by cellular transcription factors binding. Ultraviolet (UV) radiation is a major activating factor of the LCR [16]. This is due to its induction of the interferon regulatory factor (IRF)-7 expression. IRF-7 is then able to bind to HPV8 and relay the UV signal. HPV8 uses the interferon antiviral pathway for gene expression. This pathway is able to undergo suppression by IRF-3, which is another regulatory factor within the innate antiviral pathway. This discovery suggests that keratinocytes treated by IRF-3 activators might be a potential therapy against β-HPV in EV patients [17].

## 4. Acquired Epidermodysplasia Verruciformis in Iatrogenic Immunosuppressed Organ Transplant Patients

AEV has been reported in solid organ transplant recipients, particularly renal, cardiac, lung, bone marrow, and bowel transplant recipients (Table 1). The clinical onset of EV in these patients typically occurs four to seven years post-transplant and generally coincides with the prolonged immunosuppressive therapy used to prevent graft rejection [18]. This latency period demonstrates the cumulative impact of sustained immunosuppression, which gradually impairs the host’s ability to control HPV replication.

As summarized in Table 1, the majority of AEV cases have been associated with calcineurin inhibitors such as tacrolimus and cyclosporine. These calcineurin inhibitors suppress T-cell activation by obstructing interleukin-2 (IL-2) transcription and thereby impairing cytotoxic T-cell mediated clearance of HPV subtypes 5 and 8, which are most commonly associated with EV in this population [35]. This diminished immune surveillance allows for unchecked viral replication of β-HPV within keratinocytes and ultimately leads to persistent cutaneous lesions [10,14,15].

Other immunosuppressive classes also contribute uniquely to AEV development. The mammalian target of rapamycin (mTOR) inhibitors like sirolimus also suppress immune function, but mTOR inhibitors have been associated with a lower incidence of cutaneous SCC, which suggests a potential protective effect against malignant transformation. Conversely, antimetabolites such as azathioprine are known to sensitize skin to UV radiation, impair DNA repair mechanisms, and to potentially synergize with β-HPV oncogenic activity [18]. Corticosteroids are often part of baseline regimens to provide broad immunosuppression and may further compromise antiviral immune responses. However, the direct role of corticosteroids in AEV pathogenesis requires further characterization.

The development of AEV reflects the failure of cell-mediated immunity to control HPV infection. Immunosuppressive drugs reduce the activity of Langerhans cells, inhibit cytotoxic T-lymphocyte responses, and impair interferon signaling, which are key mechanisms by which the skin normally controls viral infections [36]. β-HPV types can further evade immune detection by downregulating interferon-related pathways. The downregulation of interferon-related pathways is exacerbated in immunosuppressed hosts. The impaired clearance of HPV-infected keratinocytes leads to persistent viral replication and chronic lesions in transplant recipients [37,38,39]. The degree and duration of immunosuppression, along with patient-specific factors such as age and co-morbidities, may influence the risk of developing AEV.

Clinically, AEV in transplant patients recipients presents as flat-topped, hypopigmented or erythematous papules and is often distributed across sun-exposed areas. These features often resemble those of seborrheic keratoses, viral warts, or tinea versicolor and can contribute to delayed or missed diagnoses. Histologically, lesions are characterized by enlarged keratinocytes with perinuclear halos and blue-gray cytoplasm and are consistent with viral cytopathic effects [39]. Accurate diagnosis often requires PCR-based HPV typing or immunohistochemistry to confirm the presence of EV-associated β-HPV subtypes [40].

UV radiation acts as an important cofactor in malignant pathogenesis. UV exposure induces local immunosuppression through depletion of Langerhans cells, alteration of cytokine profiles, and inhibition of interferon signaling pathways, which can further reduce the host’s ability to control viral replication [41]. Chronic UV exposure acts synergistically with β-HPV and promotes keratinocyte proliferation, which increases the risk of malignant transformation. UV exposure occurs particularly on chronically sun-exposed areas like the face and scalp. EV-associated SCCs may appear as hyperkeratotic, ulcerated, or nodular lesions and often arise within preexisting flat EV lesions. Some patients may develop multiple SCCs over time, which indicates the need for ongoing dermatologic inspection and early intervention [19,31,39].

The pathogenesis of AEV in transplant patients is fundamentally linked to immunosuppressive therapy. Immunosuppressive drugs impair T-cell mediated cytotoxicity and reduce the ability of Langerhans cells to present antigens. Immunosuppressive drugs also compromise other innate immune functions in the skin. These factors collectively facilitate persistent HPV infection and promote the development of chronic lesions [42].

Treatment strategies in these patients varies widely and includes topical retinoids, immune response modifiers, systemic retinoids, destructive procedures, and modification of immunosuppressive therapy. Because original reports have used heterogeneous outcome measures, treatment response was standardized for this review. Complete response was defined as near-total or total lesion clearance; partial response as documented clinical improvement without full resolution; no response as minimal or absent improvement; and progressive disease as lesion worsening despite therapy. Using these definitions, most transplant-associated cases demonstrated partial response or disease persistence rather than complete clearance. Follow-up duration varied, but several reports documented the development of SCC arising within or adjacent to EV lesions, underscoring the importance of long-term surveillance in this population.

## 5. Acquired Epidermodysplasia Verruciformis in Non-Transplant Iatrogenic Immunosuppressed Patients

Cases of AEV have also been reported in patients receiving iatrogenic immunosuppressive therapy who have not undergone solid organ transplantation. The cases of AEV from immunosuppressive therapy alone help support the role of impaired cell-mediated immunity in EV pathogenesis (Table 2). Each of these patients presented with phenotypic flat-topped papules on the skin after being treated with an immunosuppressive agent. Similar to transplant-associated AEV, the affected individuals lacked a family history of EV, had no consanguinity, and tested negative for HIV infection, supporting iatrogenic immunosuppression as the underlying factor. There were no reports of genetic testing conducted on the patients to determine if there was genetic predisposition to EV [43,44,45,46,47].

The cases reported in Table 2 span over a wide age range from childhood to older adulthood (4–70 years) and demonstrate comparable occurrence between sexes. The indications for immunosuppressive therapy in these patients can be categorized into autoimmune disease, hematologic malignancy, and immune dysregulation syndromes. The immunosuppressive agents administered include cyclosporine (calcineurin inhibitor), methotrexate (antimetabolite), adalimumab (TNF-ɑ inhibitor), ruxolitinib (JAK1/2 inhibitor), and bendamustine (alkylating agent). These medications share a common mechanism of impairing T-cell-mediated immune surveillance and thereby increasing susceptibility to cutaneous HPV infection and persistence [43,44,45,46,47].

Immunosuppressive agents implicated in AEV affect cell-mediated immunity through distinct mechanisms. Janus-associated tyrosine kinase-1/2 (JAK1/2) inhibitors, such as ruxolitinib, and antimetabolites, such as methotrexate, function as anti-inflammatories and emphasize the role of prolonged immunosuppression in AEV development [43,46]. Ruxolitinib plays a role in mediating host cell-mediated immune response due to the decrease in natural killer cells and cytotoxic T-lymphocytes, which further increases susceptibility to developing non-melanoma skin cancers from the impaired ability of tumor control and destruction, while methotrexate suppresses T-cell activation and proliferation [43,46]. Similarly, in bendamustine, an alkylating agent, AEV developed in parallel with progressive lymphocytopenia and established the relationship between quantitative immune deficiency and EV onset [47]. AEV reported in two patients taking adalimumab, a tumor necrosis factor (TNF-ɑ) inhibitor, is thought to have occurred potentially due to impaired cell-mediated immunity function of TNF-ɑ, which induces apoptosis in virally infected cells [45].

HPV typing was performed in a limited number of non-transplant cases with detection of EV-associated β-HPV subtypes, including HPV 5 and HPV 73 [44,45]. These findings suggest overlapping viral susceptibility profiles across different forms of immunosuppression. Histopathologic features across cases with different forms of immunosuppression were consistent with EV. The histopathologic features included acanthosis of the epidermis, hypergranulosis, and keratinocytes with perinuclear vacuolization and supported a shared clinicopathologic phenotype [43,44,45,46,47].

Therapeutic outcomes among iatrogenic AEV cases varied. Using standardized response definitions, complete resolution was reported in a minority of cases, most often following withdrawal of the immunosuppressive agent and adjunctive topical therapy. However, most cases demonstrated partial response or persistent disease despite medication discontinuation or substitution, mirroring outcomes observed in transplant-associated AEV. Methotrexate cessation led to complete lesion resolution in one patient, but most cases demonstrated disease persistence despite withdrawal or modification of immunosuppressive therapy. Disease persistence is similar to the behavior in transplant-associated AEV [45,46,47]. In contrast, a pediatric patient with atopic dermatitis developed EV lesions following cyclosporine exposure. Cyclosporine is a calcineurin inhibitor also administered to patients that developed EV post-organ transplant due to decreased T-cell activation [35,44]. This patient’s disease resolved rapidly with topical imiquimod and showed that disease severity and reversibility may vary depending on host immune recovery and therapeutic intervention. Onset times of EV lesions also varied amongst the cases. The patient treated with cyclosporine for atopic dermatitis developed papules 2 months after therapy was discontinued, but the patient who was treated with adalimumab for more than 8 years for rheumatoid arthritis did not begin developing lesions until several years into treatment. This discovery demonstrates that immune dysregulation may persist beyond active exposure to immunosuppressive agents [44,45]. These observations also suggest that the risk of AEV and its malignant sequelae are influenced not only by the class and duration of immunosuppression but also by the degree of immune dysfunction and host susceptibility. Topical agents such as imiquimod and retinoids showed variable efficacy. Surgical excision was required in refractory or malignant lesions [44,45]. Follow-up data was inconsistently reported, but SCC development was rare in non-transplant cases compared with transplant-associated AEV, although limited follow-up precludes definitive conclusions. The persistence of lesions despite treatment and therapy adjustment suggests that immune reconstitution alone may be insufficient for disease clearance once HPV persistence is established.

Collectively, these non-transplant AEV cases emphasized that this disease represents a broader consequence of sustained immune dysfunction instead of a transplant-specific complication. Awareness of AEV across diverse immunosuppressive contexts is critical because early identification and intervention may reduce the risk of chronic disease progression and development of non-melanoma skin cancer.

## 6. Treatment of Acquired Epidermodysplasia Verruciformis

Treating AEV in transplant recipients remains challenging, as current therapeutic options are limited in efficacy and are not standardized. Current treatment strategies are largely extrapolated from inherited EV and have variable outcomes. Due to the increase in non-melanoma skin cancer in the transplant population, regular dermatologic inspection, early biopsy of suspicious lesions, and sun protection are essential means to control active lesions and prevent malignant transformation in both transplant and non-transplant AEV patients.

Topical agents are the most commonly reported first-line AEV interventions in the literature. As summarized in Table 1 and Table 2, multiple treatment strategies have been attempted, including retinoids, immune response modifiers, and keratolytics, which resulted in variable success. Despite these efforts, complete lesion clearance has been reported in only a small subset of cases. One patient achieved resolution with weekly application of 0.5% tazarotene cream, while another responded to a multimodal regimen involving topical imiquimod, topical tretinoin, oral acitretin, and three doses of the 9-valent HPV vaccine (Gardasil) [30,33]. Similarly, Martinez-Molina et al. described the use of the nonvalent HPV vaccine resulting in clinical improvement [34]. These findings are notable because currently available HPV vaccines primarily target mucosal high-risk α-HPV subtypes (6, 11, 16, 18, 31, 33, 45, 52, 58) rather than the β-HPV subtypes classically associated with EV. The observed benefit may therefore reflect indirect immune stimulation, cross-protective immune responses, or enhancement of HPV-specific cellular immunity rather than direct antiviral activity. Further investigation into first-line AEV is warranted. Even the therapeutic response to HPV vaccination has not been uniform. Orellana-Westermeyer et al. reported that lesions were only partially cleared despite a combined approach that involved vaccination (Gardasil 9). Immunosuppressant adjustment to everolimus and topical retinoic acid suggested that treatment efficacy may depend on host immune status, degree of immunosuppression, and viral subtype [24].

Since immunosuppression plays central role in the pathogenesis of acquired epidermodysplasia verruciformis, modification of immunosuppressive therapy becomes an important therapeutic consideration. In transplant recipients, the primary objective is to reduce lesion development while maintaining adequate graft function and minimizing the risk of rejection. In non-transplant patients receiving iatrogenic immunosuppression for autoimmune disease or malignancy, treatment modification must similarly balance disease control with restoration of effective cell-mediated immunity. Although strategies such as dose reduction, temporary discontinuation, or substitution of immunosuppressive therapy have been attempted in both populations, reports indicate that AEV lesions may persist despite these adjustments [20,24,36,47]. This finding suggests that persistent disease may result from complex interactions between viral latency, host immunity, and skin microenvironment [7].

## 7. Discussion

AEV represents a cutaneous manifestation of impaired cell-mediated immunity. Historically, it has been reported primarily in the context of HIV infection or post-solid organ transplantation. However, the cases summarized in this review demonstrate that AEV can develop across a broad spectrum of iatrogenic immunosuppressive states, which include treatment with calcineurin inhibitors, antimetabolites, biologic agents, tyrosine kinase inhibitors, and cytotoxic chemotherapy [43,44,45,46,47]. This spectrum of multiple therapies reinforces that AEV is not transplant-specific but rather reflects a shared consequence of immune dysregulation in which compromised cutaneous immune defense permits persistent β-HPV infection and lesion development.

Comparison of transplant an non-transplant immunosuppressed populations provides several useful insights. Transplant recipients most commonly develop AEV in the setting of long-term multidrug immunosuppression, often involving calcineurin inhibitors and antimetabolites, whereas non-transplant cases more frequently occur with targeted immunomodulatory therapies or systemic treatments for autoimmune or malignant conditions. Despite these differences in underlying disease and treatment regimens, the clinical morphology, HPV subtypes, and histopathologic findings reported across groups are largely similar. This overlap suggests that diverse immunosuppressive mechanisms converge on a common pathway of impaired cutaneous viral control. The comparison also highlights that cumulative immune suppression, rather than a single medication class, is likely the major driver of disease development.

Across both transplant and non-transplant populations, AEV occurs in patients without a family history of epidermodysplasia verruciformis and develops disease later in life, which strongly supports an acquired defect in antiviral immunity. The β-HPV subtypes most frequently associated with AEV, including HPV 5 and HPV 8, are generally considered commensal in immunocompetent hosts [15,16]. In immunosuppressed individuals, these viruses gain the ability to replicate unregulated, which leads to chronic cutaneous lesions. Unlike α-HPV infections, which are typically cleared efficiently, β-HPV relies heavily on compromised cell-mediated immunity [15]. The pattern of infection underscores the importance of immune surveillance in limiting viral pathogenicity.

Immunosuppressive agents implicated in AEV share pathways that impair antiviral defense even though each class acts through distinct mechanisms. Implicated agents reduce T-cell-mediated cytotoxicity, disrupt cytokine signaling, and impair innate immune responses critical for controlling β-HPV infection across both transplant and non-transplant populations. This supports the interpretation that cumulative immune dysfunction, rather than exposure to a single medication class, is the primary determinant of disease development. Interactions between viral latency and incomplete immune reconstitution may account for the persistence of AEV lesions even after partial reduction or withdrawal of immunosuppressive therapy.

Management of AEV remains challenging, and is largely individualized, and complicated by the variable efficacy of available therapies. Topical retinoids, immune response modifiers, systemic retinoids, and HPV vaccination have demonstrated partial success, but these therapies are often limited to lesion regression rather than clearance (Table 1 and Table 2). Clinical improvement following HPV vaccination has been reported despite lack of direct coverage against β-HPV subtypes. This suggests that there is indirect immune stimulation, cross-protective responses, or enhancement of HPV-specific cellular immunity. However, the mechanism underlying this response remains speculative. Variability in treatment outcomes reflects differences in residual immune function, viral load, β-HPV subtype, and the degree of host immunosuppression. Patients with prolonged immunosuppression often achieve only partial clearance, which could indicate that enhancing host antiviral immunity is as critical as pharmacologic intervention.

AEV has clinical significance due to its association with non-melanoma skin cancers, especially cutaneous SCC. Increased SCC risk in immunosuppressed patients with EV is well supported by observational data, especially among transplant recipients. Ultraviolet radiation is also a recognized contributor to cutaneous carcinogenesis and may act as a cofactor by promoting local immune suppression and keratinocyte DNA damage. While interactions between β-HPV persistence, UV exposure, and malignant transformation have been proposed, the precise mechanistic relationships remain incompletely defined.

## 8. Clinical Takeaways

Clinicians should suspect AEV in immunosuppressed patients who develop persistent, flat-topped, wart-like lesions, particularly when lesions are widespread, refractory to standard therapies, or localized to sun-exposed areas. Diagnostic evaluation should include skin biopsy to confirm characteristic histopathologic features, with HPV typing performed when feasible to identify EV-associated β-HPV subtypes.

Given the potential risk of malignant transformation, patients with AEV benefit from regular dermatologic surveillance, patient education regarding photoprotection, and prompt evaluation of evolving or suspicious lesions. Awareness of AEV across both transplant and non-transplant immunosuppressive contexts is important, as early recognition and surveillance may reduce the risk of chronic disease progression and cutaneous squamous cell carcinoma.

## 9. Conclusions

AEV is a rare but clinically significant complication of immunosuppression and is driven by β-HPV infection in the context of impaired cell-mediated immunity. The emergence of AEV in transplant and non-transplant populations highlights a broader model of viral persistence under immune compromise, showing that AEV reflects cumulative immune dysfunction across iatrogenic immunosuppression. Management of AEV remains challenging, with variability in treatment responses demonstrating the need for therapies that enhance antiviral immune function. The predominance of case reports, inconsistent HPV typing, limited assessment of genetic susceptibility, and heterogeneous outcome reporting limit the interpretation of long-term risk and therapeutic efficacy. Future studies should focus on targeted immunomodulatory interventions, prospective monitoring, and standardized outcome measures to establish evidence-based management strategies and reduce the risk of malignant transformation in immunosuppressed populations.

## Figures and Tables

**Table 1:** Reported cases of AEV in transplant recipients.

Author	Sex	Age (Year)	Transplant Type	Immunosuppressant	HPV Type	Treatment	Outcomes
Lutzner et al. [19]	M	33	Renal	Corticosteroids and azathioprine	HPV 5		Bowenoid carcinoma (SCC)
	F	23	Renal	Corticosteroids and azathioprine	HPV 3, 5		
Hirschman et al. [20]	F	3	Small bowel	Tacrolimus and sirolimus	HPV 14	Reduction of tacrolimus dose	
Mendes et al. [21]	M	24	Renal	Sirolimus, mycophenolate sodium, and prednisone			
Kinariwalla et al. [22]	M	14	Cardiac	Tacrolimus and azathioprine		Tretinoin	
	M	7	Cardiac	Sirolimus, azathioprine, prednisone, and tacrolimus			
	M	12	Cardiac	Sirolimus, azathioprine, prednisone, and tacrolimus		Tretinoin, cidofovir, and glycolic acid	
Gale et al. [23]	M	46	Renal	Methylprednisolone, mycophenolate mofetil, and tacrolimus	HPV 20	Cryotherapy and imiquimod	
Orellana-Westermeyer et al. [24]	F	39	Renal	Prednisone and tacrolimus		Everolimus, imiquimod, HPV (Gardasil 9) vaccine, and topical retinoic acid	Partial lesion clearance
Gomez-Bernal et al. [25]	F	19	Renal	Prednisone, tacrolimus, and mycophenolate mofetil	HPV 23	Corticosteroids and antifungals	
Gara et al. [26]	M	30	Renal	Prednisone, mycophenolate mofetil, and tacrolimus			
Henley et al. [27]	F	44	Renal	Cyclosporine, mycophenolate mofetil, and prednisone		Cryosurgery, tazarotene, imiquimod, and 5-fluorouracil	
di Prinzio et al. [28]	F	39	Renal	Meprednisone and tacrolimus			
Alturo-pons et al. [29]	M	40	Bone marrow and lung	Tacrolimus, methylprednisolone, and polychemotherapy			Oral SCC
Kunishige et al. [30]	M	33	Peripheral blood stem cell	Tacrolimus	HPV 8, 20	Tazarotene	
Hopfl et al. [31]	M	38	Renal	Azathioprine and prednisolone	HPV 38, RTRX1, ICPX1	Cryotherapy, surgical resection, and 5-fluorouracil	SCC, Basal Cell Carcinoma, Bowen’s disease, and solar keratoses
Cravero et al. [32]	F	67	Renal	Tacrolimus, mycophenolate, and prednisone			
Maor et al. [33]	F	50	Renal	Tacrolimus, mycophenolate, and prednisolone	HPV 5	Gardasil vaccination, imiquimod, tretinoin, and oral acitretin	Complete lesion clearance
Martinez-Mollina et al. [34]	F	59	Renal	Tacrolimus, prednisone, and mycophenolate mofetil		Nonvalent HPV vaccine	Complete lesion clearance

**Table 2:** Reported cases of AEV in iatrogenic immunosuppression (non-transplant).

Author	Sex	Age (Year)	Indication	Immunosuppressant	HPV Type	Treatment	Outcomes
Demirel Ogut and Mizrak [43]	F	67	Polycythemia vera	Ruxolitinib		Acitretin and topical tazarotene (ineffective)	SCC
Fernandez et al. [44]	F	4	Atopic dermatitis	Cyclosporine	HPV 5	Topical pimecrolimus (ineffective) and topical imiquimod (effective)	Complete lesion clearance
Schultz et al. [45]	M	70	Rheumatoid arthritis	Adalimumab	HPV 73	Discontinuation of adalimumab and topical imiquimod (ineffective), surgical excision (effective)	Complete lesion clearance
	M	68	Psoriasis	Adalimumab			
AlFada and AlHumidi [46]	M	23	Recalcitrant disseminated granuloma faciale	Methotrexate		Discontinuation of methotrexate (effective)	Complete lesion clearance
Cougoul et al. [47]	F	40	Hodgkin lymphoma	Bendamustine		Discontinuation of bendamustine (partially effective)	Partial lesion clearance

## Data Availability

No new data were created or analyzed in this study.

## Abbreviations

AEV	acquired epidermodysplasia verruciformis
EV	epidermodysplasia verruciformis
HIV	human immunodeficiency virus
HPV	human papilloma virus
IL	interleukin
IRF	interferon regulatory factor
LCR	long control region
mTOR	mammalian target of rapamycin
NCR	non coding region
SCC	squamous cell carcinoma
UV	ultraviolet
